# Postoperative Delayed Duodenum Perforation following Elective Laparoscopic Cholecystectomy

**DOI:** 10.1155/2014/823149

**Published:** 2014-03-25

**Authors:** Kong Jing, Wu Shuo-Dong

**Affiliations:** Department of the First Minimal Invasive Surgery and Bile Duct Surgery, Sheng Jing Hospital of China Medical University, Shenyang, Liaoning 110004, China

## Abstract

*Background*. Duodenum injury is extremely rare complication of laparoscopic cholecystectomy with potentially fatal consequences. *Methods*. Over the past 13-year period, 3000 laparoscopic cholecystectomies were performed in our institution. Duodenum injury only occurred in one patient recently who had undergone gastrectomy owing to duodenal diverticulum. The diagnosis and management of this rare complication of laparoscopic cholecystectomy are described, and the literature is reviewed. *Results*. We present this case of duodenum injury on the fourth postoperative day after selective laparoscopic cholecystectomy was treated successfully by percutaneous needle aspiration and catheter drainage. The hospital stay was 26 days. No abscess recurred during the follow-up period. *Conclusion*. Duodenum injuries are extremely rare complications of laparoscopic cholecystectomy with potentially fatal consequences if not promptly recognized and treated. Sonographically guided percutaneous needle aspiration and catheter drainage can be used to treat the intraperitoneal abscess. Billroth II subtotal gastrectomy and gastrojejunostomy were beneficial for the treatment.

## 1. Introduction

Laparoscopic cholecystectomy (LC) has become the standard approach in the treatment of benign gallbladder disease. It may lead to serious complications, some of which can be disastrous if they are not recognized and managed immediately. Duodenum injury is extremely rare complication of laparoscopic cholecystectomy with potentially fatal consequences. It was commonly unrecognized at the time of procedures and was diagnosed later when the patients experienced sepsis, peritonitis, intraperitoneal abscess, or enterocutaneous fistula. Over the past 13-year period, 3000 laparoscopic cholecystectomies were performed in our institution. Duodenum injury only occurred in one case recently. Herein we present this case of duodenum injury secondary to selective laparoscopic cholecystectomy presenting as fever and abdominal abscess on the fourth postoperative day. He was treated successfully with sonographically guided percutaneous needle aspiration and catheter drainage. The diagnosis and management of this rare complication of laparoscopic cholecystectomy are described, and the literature is reviewed.

## 2. Case Report

A 74-year-old man with mild right upper quadrant pain for 2 years was admitted to the hospital for acute onset of severe upper abdominal pain radiating to the right shoulder. His medical history was remarkable, expect for subtotal gastrectomy 20 years ago owing to diverticulum at duodenal bulb. His vital signs and physical examination were normal. Laboratory data at presentation were as follows: white blood cells 5.9 × 10^9^/L (reference 3.6–9.7 × 10^9^); hemoglobin 13.0 g/dL (reference 12–16); alanine aminotransferase 28 *μ*/L (reference 0–40); direct bilirubin 4.3 *μ*mol/L (reference 0–8.6); and alkaline phosphatase 63 *μ*/L (reference 40–150). Abdominal ultrasonography (US) demonstrated one big stone with 2.5 × 1.7 cm in the gallbladder, and the thickness of gallbladder wall was 0.4 cm. No evidence was seen of biliary obstruction.

**Figure 1:**
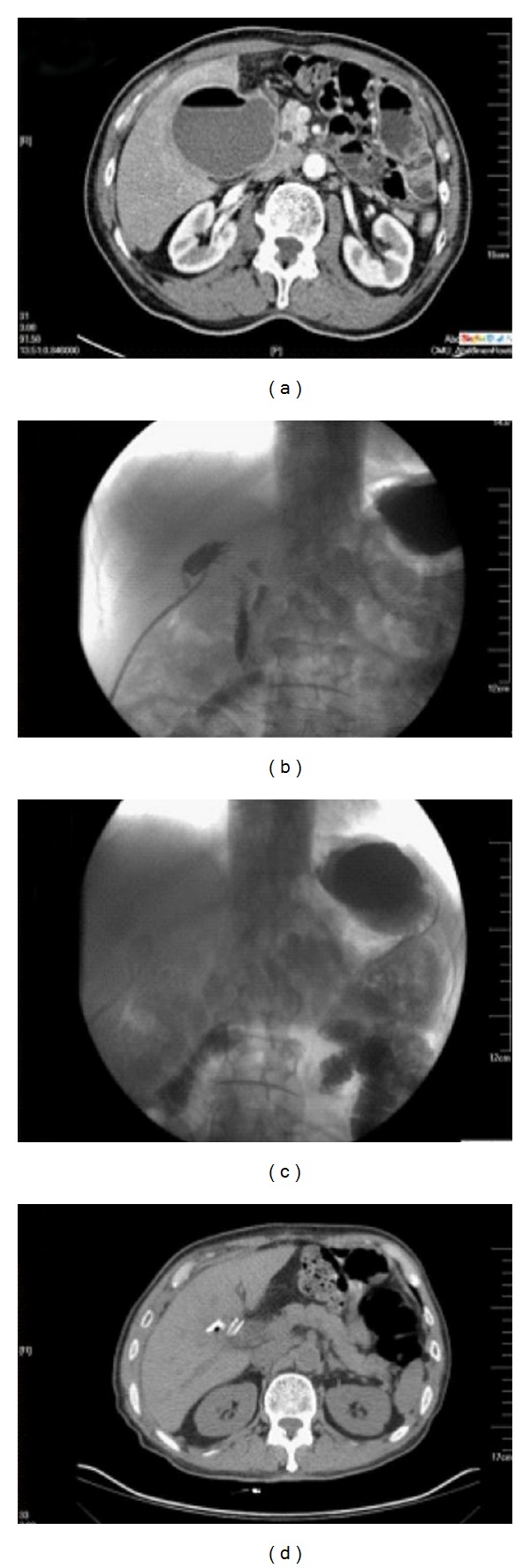
(a) A cyst-mass in the area of hepatic portal pneumonia was seen through abdominal CT. (b) When contrast agent was injected from the catheter, it was found to flow into the duodenal stump on the image. (c) When gastrointestinal X-ray was performed, contrast agent in stomach flow into jejunum directly. (d) Abdominal CT demonstrated that the cyst-mass in the area of hepatic portal disappeared.

The patient underwent selective laparoscopic cholecystectomy successfully. The operation lasts for 55 minutes. The patient resumed food intake on postoperative day 2. The temperature was elevated between 37.5°C and 38.6°C from the postoperative day 4. Abdominal Computerized Tomography (CT) demonstrated a cyst-mass in the area of hepatic portal (see Figure 1(a)). Sonographically guided percutaneous needle aspiration was performed. During the course, contrast agent was found to flow into the duodenal stump directly and duodenal stump leakage was confirmed (see Figure 1(b)). Gastrointestinal X-ray showed that contrast agent in stomach flow into jejunum directly and this demonstrated that Billroth II subtotal gastrectomy and gastrojejunostomy have been done in this patient (see Figure 1(c)). The catheter drainage was kept. The patient was treated conservatively with fasting and nutritional support for one week. He did not complain of peritonitis and fever after that. And then a liquid diet was taken again. The catheter was closed two weeks later. Abdominal CT demonstrated that the cyst-mass disappeared (see Figure 1(d)). Then the catheter was removed. He was discharged from the institution and the hospital stay was 26 days. There was no abscess recurred during the follow-up period for one year.

## 3. Discussion

Laparoscopic cholecystectomy (LC) is the standard treatment for symptomatic cholelithiasis. Growing experience with various laparoscopic techniques and the rapid advance in instrumentation have led to the use of the procedure not only electively but also in complicated gallstone disease or in patients with previous abdominal surgery with extensive adhesiolysis.

Duodenum injuries are extremely rare complications of laparoscopic cholecystectomy with potentially fatal consequences if not promptly recognized and treated. It was reported more frequently during 1990s when laparoscopic cholecystectomy was at its initial stage. In Marakis GN's report, 1225 laparoscopic cholecystectomies were performed and duodenum injury happened in one case (0.08%) [1]. Duodenal perforations were usually caused by the improper use of irrigator-aspirator device, or by electrosurgical and laser burns, or compressed by Titanium clips [2]. Over the past 13-year period, 3000 laparoscopic cholecystectomies were performed in our institution. Duodenum injury only occurred in one patient who had undergone gastrectomy owing to duodenal diverticulum in 2010. The case in our report was asymptomatic during the immediate postoperative period. We had described a presumed conductive burn injury of the posterior second portion of the remaining duodenal diverticulum during retracting the duodenum. However, during dissection in the triangle of Calot, the remaining duodenal bulb diverticulum is at risk for direct contact burn or energy conduction burn. This unrecognized injury resulted in full-thickness necrosis of the thin wall of duodenal bulb with delayed perforation. In order to avoid the electrothermal injury of electrosurgical generator we often use ultrasound scalpel during the laparoscopic surgery. But the special history and anatomical abnormalities should be paid more attention during the operation.

Duodenum injuries are extremely fatal consequences if not promptly recognized and treated. El-Banna et al. reported four duodenal injury cases in 2000, three of them died [3]. Patients with a perforated duodenum usually present with an acute abdomen pain and more than 95% require emergency operative intervention. Testini et al. reported their experience of managing 5 patients affected by descending duodenal injuries secondary to laparoscopic cholecystectomy [4]. In all cases an emergency laparotomy was done. Two patients underwent direct suture of the duodenum and external biliary drainage through a T-tube; 1 case underwent a duodenojejunostomy and another duodenopancreatectomy.

In our report, this patient was successfully managed without laparotomy and the total costs of this patient were little more than those with no complications. It was due to the following factors. Firstly, delayed perforation of a duodenum creates a full-thickness hole, but free spillage is prevented by creation of a walled-off area with contiguous organs. The widespread peritonitis was not complained in this patient. Secondly, he has undergone Billroth II subtotal gastrectomy. Oral food does not transit through the duodenum and the healing of the duodenal perforation was not affected. Third, perforation in the duodenal cap had a better result than that at the descending duodenum or the duodenal papilla. And the latter is a more complicated anatomical position which can be affected by pancreatic fluid and bile severely.
